# Perinatal emotional skills groups for women and birthing people with borderline personality disorder: outcomes from a feasibility randomised controlled trial

**DOI:** 10.1192/bjo.2024.833

**Published:** 2024-12-26

**Authors:** Paul Moran, Debra Bick, Lucy Biddle, Belinda Borries, Rebecca Kandiyali, Farah Mgaieth, Vivan Patel, Janice Rigby, Penny Seume, Vaneeta Sadhnani, Nadine Smith, Michaela Swales, Nicholas Turner

**Affiliations:** Centre for Academic Mental Health, Population Health Sciences Department, Bristol Medical School, University of Bristol, UK; Warwick Clinical Trials Unit, Warwick Medical School, University of Warwick, UK; Population Health Sciences Department, Bristol Medical School, University of Bristol, UK; Specialist Community Perinatal Mental Health Service, Avon & Wiltshire Mental Health Partnership NHS Trust, Bristol, UK; Centre for Health Economics, Warwick Clinical Trials Unit, University of Warwick, UK; Department of Clinical, Educational & Health Psychology, University College London, UK; Channi Kumar Mother and Baby Unit, Bethlem Royal Hospital, South London and Maudsley NHS Foundation Trust, London, UK; Patient and Public Involvement and Engagement Lead, University of Bristol, UK; North Wales Clinical Psychology Programme, Bangor University, UK

**Keywords:** Borderline personality disorder, perinatal psychiatry, randomised controlled trial, psychological treatments, personality disorders

## Abstract

**Background:**

There is no clear evidence about how to support people with borderline personality disorder (BPD) during the perinatal period. Perinatal emotional skills groups (ESGs) may be helpful, but their efficacy has not been tested.

**Aims:**

To test the feasibility of conducting a randomised controlled trial (RCT) of perinatal ESGs for women and birthing people with BPD.

**Method:**

Two-arm parallel-group feasibility RCT. We recruited people from two centres, aged over 18 years, meeting DSM-5 diagnostic criteria for BPD, who were pregnant or within 12 months of a live birth. Eligible individuals were randomly allocated on a 1:1 ratio to ESGs + treatment as usual (TAU), or to TAU. Outcomes were assessed at 4 months post randomisation.

**Results:**

A total of 100% of the pre-specified sample (*n* = 48) was recruited over 6 months, and we obtained 4-month outcome data on 92% of randomised participants. In all, 54% of participants allocated to perinatal ESGs attended 75% of the full group treatment (median number of sessions: 9 (interquartile range 6–11). At 4 months, levels of BPD symptoms (adjusted coefficient −2.0, 95% CI −6.2 to 2.1) and emotional distress (−2.4, 95% CI −6.2 to 1.5) were lower among those allocated to perinatal ESGs. The directionality of effect on well-being and social functioning also favoured the intervention. The cost of delivering perinatal ESGs was estimated to be £918 per person.

**Conclusions:**

Perinatal ESGs may represent an effective intervention for perinatal women and birthing people with BPD. Their efficacy should be tested in a fully powered RCT, and this is a feasible undertaking.

**Trial registration:**

ISRCTN80470632.

Borderline personality disorder (BPD) is a common mental disorder associated with enduring psychosocial impairment and a suicide rate that is 45 times higher than in the general population.^1^ The perinatal period poses particularly difficult challenges for women and birthing people diagnosed with BPD. They are more likely to experience adverse pregnancy outcomes, including higher rates of gestational diabetes, preterm birth and delivering babies with lower Apgar scores.^2^ Following childbirth, they may experience high levels of distress precipitated by the increased demands associated with having a baby.^3^ Furthermore, their children are at increased risk of psychological problems and of being taken into care.^4–6^ Yet, currently there is no clear evidence about how to help them during the perinatal period.^7^

## Supporting people with BPD during the perinatal period

Currently in the UK, people with BPD receive standard perinatal mental healthcare, consisting of an assessment, care plan and regular reviews.^8^ However, this fails to account for the complexity of needs associated with BPD and may be associated with iatrogenic harm, including off-licence prescribing of medication.^9^ BPD is treatable using structured psychological treatment, such as dialectical behaviour therapy (DBT).^10^ However, the current trial evidence is derived exclusively from non-perinatal populations. In addition, treatments such as DBT are lengthy and expensive to deliver and geographically, access to such interventions is extremely variable.^11^ DBT includes both individual and group work; the group work consists of facilitated skills-training groups. These groups teach individuals how to deal more effectively with emotions and relationships, and when delivered as a stand-alone intervention, they may help people with BPD,^12^ although definitive evidence of efficacy is lacking. A recent systematic review^12^ identified three small randomised controlled trials of DBT-informed skills groups for non-perinatal populations which showed evidence of moderate effect, considered clinically important, for BPD severity (standardised mean difference, SMD −0.66, 95% CI −1.08 to −0.25, *n* = 3 studies, *n* = 184 participants) and psychosocial functioning (SMD −0.45, 95% CI −0.75 to −0.16, *n* = 3 studies, *n* = 184 participants). Yet none of the studies included perinatal populations, and the overall quality of the evidence derived from these three trials was graded as low.

DBT skills groups have been adapted for a variety of clinical populations, including ‘high-risk’ mothers,^13^ as well as for people with BPD during the perinatal phase.^14^ Emotion regulation skills groups (ESGs) can be delivered online (thereby increasing access to treatment for those in remote locations) and are popular with patients and clinicians working in perinatal mental health teams. However, there is no robust evidence to support the efficacy of perinatal ESGs. Testing this would require conducting a fully powered clinical trial, yet it is unclear whether such a trial is feasible. Recruitment and retention of participants into clinical trials is always challenging,^15^ and these challenges are likely to be compounded during the perinatal period, when there are additional physiological and psychological demands on potential participants. In this paper, we report findings from a study investigating the feasibility of conducting a randomised controlled trial (RCT) of perinatal ESGs, as an adjunct to treatment as usual (TAU) versus TAU alone for people with BPD.

## Method

### Study design

We conducted a two-arm parallel group feasibility RCT with a 16-week follow-up (trial registration: ISRCTN 80470632). A nested qualitative evaluation was embedded within the trial, to enhance insight into participants’ experiences of the study and the intervention. A more detailed description of the protocol has been published previously.^16^

### Setting and participants

Study participants were recruited from one perinatal mental health service in South East London and one service in South West England. Both services included areas of high deprivation, ethnic diversity and high rates of psychiatric morbidity. Within each service, the principal investigator was asked to seek the consent of potentially eligible people to be contacted for screening into the study. To take part, potential participants had to be aged 18 years or over; either pregnant (from week 15 of gestation onwards) or within 12 months of having a live birth; and likely to have a diagnosis of BPD.

We excluded people not meeting screening criteria for BPD (see the screening assessment list in the next section) as well as the following groups:

- Those with a diagnosis of a coexisting organic, psychotic mental disorder or substance use dependence syndrome.
- Those with cognitive or language difficulties that would preclude them from providing informed consent or compromise participation in study procedures. Participants also had to have good command of English to participate in the study. This was because, for those randomised to perinatal ESGs, it was not possible to run synchronous multilingual online groups. In addition, we were limited to carrying out assessments in English, as the outcome scales had not all been translated or validated in other languages.
- Those posing an acute risk to their baby, as assessed by clinicians.
- Those requiring admission to a mother and baby unit.
- Those who did not have internet access, as this was a requirement to access the intervention.

If the principal investigator believed that an individual was likely to meet the eligibility criteria, they provided verbal and written information about the study and determined whether the person agreed to being contacted by a member of the research team. Individuals were offered a copy of a patient information sheet which explained that if they agreed to share their details with the research team, the initial step would involve participating in a telephone or online screening interview. The contact details and preferred method of contact were then passed on to the trial manager, who arranged a time to conduct a screening interview with individuals who consented to be contacted.

### Screening assessment of potential participants

The screening assessment consisted of three elements:

- The self-report Standardised Assessment of Personality Abbreviated Scale (SAPAS)^17^ to assess for the presence of probable personality disorder. We excluded people who scored less than 3 on the SAPAS.
- The researcher also completed a 15-item BPD checklist with the person,^18^ confirming the presence or absence of DSM symptoms of BPD. In keeping with diagnostic guidelines,^19^ participants needed to positively endorse at least 5/9 of the symptom domains covered by the 15 items to be eligible for the trial.
- The researcher checked whether the individual was willing and able to receive perinatal ESGs if they were randomised to this.

Following the eligibility check and prior to commencing the baseline assessment, the researcher obtained the written consent of all eligible people relating to their participation in the trial. Consent was obtained via an online e-consent method, and participants were emailed an electronic copy of their e-consent form.

### Interventions

Those in the intervention arm of the trial were offered perinatal ESGs in addition to standard perinatal mental healthcare. Participants receiving ESGs continued to be cared for as usual by their perinatal mental health team. Perinatal ESGs were delivered online as specified in the Maternal Emotional Wellbeing manual.^20^ The intervention comprises two 1-h individual preparatory sessions, followed by twelve 2-h group sessions. The individual preparatory sessions involved establishing specific, individually tailored, behavioural-specific goals for the group intervention, and increasing motivation to attend and apply the skills to daily life, as well as an opportunity to meet the facilitators and ask questions. Groups were organised into three modules consisting of emotion regulation, distress tolerance and interpersonal effectiveness, with mindfulness components throughout. Each session was supplemented with ‘keeping baby in mind’ which focused on utilising the skills as a parent of a new child, with reflections on child development. Mindfulness is a foundation skill that supports the individual to become aware of thoughts, feelings, urges and body sensations in a non-judgemental way as they are occurring. Emotional regulation depends on the ability to name emotions and assess if they ‘fit the facts’ of a particular situation to then prepare for appropriate emotional expression and problem-solving. Emotional regulation skills also include skills to increase emotional resilience, such as sleep hygiene, regular exercise and a healthy diet. Many of these lifestyle domains change significantly during the transition into parenting, making this a particularly important topic to cover at this time. Distress tolerance skills are designed to support skilful survival of challenging times when immediate problem-solving is not possible. The final set of skills explore interpersonal effectiveness, making new friends, and expressing and making requests in skilful ways that keep relationships and self respect. The transition into early years parenting typically involves significant changes in friendship groups and handling new social scenarios. Each group session included time to reflect and review the application of the skills in daily life to support the strengthening and generalisation of the skills.

Participants were constantly encouraged to consider how their application of skills would impact on their interactions with their babies (born and not yet born). Each DBT skill session dedicated a minimum of 15 min at the end of each class where application of the skill to infants and the parent–infant relationship was discussed and rehearsed. In the general teaching component of the class (45 min), skills trainers utilised examples relevant to the perinatal period to teach the essential elements of the skills. In the homework review component (1 h), participants were encouraged to discuss issues in relation to their infants, their parental role and their support structures (partner and familial relationships).

The aim of the intervention was to assist participants to learn and implement skills to address challenges in their everyday life, and so the focus was on the acquisition and strengthening of emotional skills in the context of the typical day-to-day experience of looking after an infant. Although there was a teaching component, participants were actively encouraged to speak, and react to and validate other participants’ efforts. In addition, multiple strategies were utilised to optimise engagement in the online group work. In the individual preparatory meetings, each participant was asked to reflect on strategies they might need to use to stay engaged with group discussion, particularly if their baby needed soothing or there were other distractions. In the first session, rules and expectations were clearly laid out, including the need to always keep video cameras on and work hard with active listening. In the spirit of dialectics, facilitators also validated the challenge of juggling child care alongside participating in the groups. Finally, homework and skills practice tasks were assigned each week, and this (along with group feedback from other participants) provided facilitators with an opportunity to monitor individuals’ engagement with skills development each week.

The groups were run by a clinical psychologist working with another qualified perinatal clinician. All therapists were employed by the local NHS Trusts for the duration of the study. Staff were trained to deliver perinatal ESGs through a 2-day online workshop led by co-author M.S. who is a recognised trainer in DBT. Once a week, an online Microsoft Teams consultation meeting was held between recruiting centres, to promote sharing of good clinical practice through peer monitoring and encouragement. The group facilitators monitored and recorded the participants’ ESG attendance using Excel spreadsheets which were shared with the trial manager at the end of treatment. Participants who missed three consecutive ESG sessions were considered to have dropped out of treatment.

TAU was standard perinatal mental healthcare, delivered on an individual basis by staff working in the specialist perinatal community mental health team. It was delivered in accordance with current National Institute for Health and Care Excellence (NICE) and Royal College of Psychiatry guidelines,^21^ and consisted of assessment, a written care plan and regular reviews with a care coordinator. In addition to assessment and regular reviews with a care coordinator, standard care could include individual therapy, occupational therapy, nursery nurse interventions and video interaction guidance. We did not restrict access to these therapies. However, participants randomised to TAU were not able to access other group-based interventions providing related emotional skills training.

### Randomisation

After consenting to participation and completing assessments, eligible individuals were randomly allocated on a 1:1 ratio to either perinatal ESGs + TAU or to TAU alone. The randomisation sequence was generated by the Research Electronic Data Capture Service (REDCap) at the University of Bristol (https://brtcclinical.bris.ac.uk/redcap/). Randomisation was stratified by centre. Throughout the study, the randomisation list was encrypted and held with the trial coordinating office. The trial manager informed participants and clinical teams of the participants’ allocation status. It was not possible to blind participants or treating clinicians to the participants’ treatment allocation because of the nature of the intervention; however, the statistician and health economist were blind to the trial condition throughout the study.

### Measures

At baseline, we collected the following sociodemographic information on all participants: relationship status, age, ethnicity, highest education level attained, living arrangements and current employment status. They were also asked about whether they were in the antenatal or postnatal period.

Study participants completed the following outcome measures at baseline and again at 2 and 4 months post randomisation:

- Zanarini Rating Scale for Borderline Personality Disorder Self-Report Scale (ZAN-BPD)^22^ – a validated measure of symptoms experienced by people diagnosed with BPD over the past week.
- Clinical Outcomes in Routine Evaluation (CORE-10)^23^ – a brief measure of psychological distress.
- Health-related quality of life was assessed using the EuroQol-5 Dimensions-5 Levels (EQ-5D-5L) – a descriptive system and a Visual Analogue Scale (VAS) for health-related quality of life states in adults.^24^
- Short Warwick-Edinburgh Mental Wellbeing Scale (SWEMWBS) – a reliable^25^ 7-item scale of mental well-being covering subjective well-being and psychological functioning.
- Work and Social Adjustment Scale (WSAS)^26^ – a simple, reliable and valid measure of impaired functioning that is sensitive to change.
- Parenting Stress Scale (PSS)^27^ – a self-report measure of an individual's feelings about positive and negative aspects of parenthood..
- Self-harming behaviour was assessed using a single question: ‘Have you [in the past week] deliberately taken an overdose (e.g. of pills or other medication) or tried to harm yourself in some other way (such as cut yourself)?’^28^
- A resource questionaire to examine patterns of health and social care service use.

### Qualitative evaluation

After completing treatment and all quantitative follow-up assessments, a purposive sample of participants was invited to participate in individual semi-structured interviews. Sixteen trial participants and five therapists completed these interviews at the end of the intervention. The sample included participants from both arms of the trial (intervention arm: *n* = 10; TAU: *n* = 6), as well as individuals who were pregnant (*n* = 5) and those who were postnatal (*n* = 11) at recruitment. The interviews were audi-recorded and facilitated using a topic guide. Trial participant interviews focused on their views about the acceptability of study procedures and experiences of treatment. Staff interviews focused on the research process, the value of training and supervision, and the potential effects of perinatal ESGs. Consenting trial participants were offered a £20 voucher to reimburse their time and effort for completing the qualitative interviews.

### Adverse events

All adverse events were assessed for seriousness, causality and expectedness by the principal investigator, and recorded and reported from the point of consent (baseline assessment) until the 4-month follow-up assessment or the point of withdrawal from the study. Hospitalisations for elective treatment of a pre-existing condition were not reported as serious adverse events.

### Data management

Web-based electronic case report forms were used to collect baseline and outcome data. No data analysis was undertaken until the databases were locked. Trial staff ensured that the participants’ anonymity was maintained through protective, secure handling and storage of participant information at the trial centres. All data and study documents were stored on a secure database at the university coordinating centre.

### Data analysis

In keeping with recommendations for feasibility studies,^29^ we did not base plans for sample size on a power calculation. We judged that a sample size of 48 would allow us to estimate, with a desirable degree of precision, the rate of recruitment and retention in a future definitive trial. Our criteria for determining the success of the feasibility study were recruitment of at least 36 participants (75% of the target sample), uptake of treatment (predefined as 75% of sessions) by at least 75% of participants in the intervention arm, and completion of 4-month follow-up assessments by 75% of the target sample.

Since this was a feasibility trial, the study was not designed to detect statistically significant differences between the two groups. Therefore, the primary analyses were descriptive, concentrating on participant characteristics, completeness of follow-up data and adherence to treatment. Data were analysed and reported following the Consolidated Standards of Reporting Trials (CONSORT) guidance extension to feasibility studies,^30^ including a CONSORT flow diagram. Despite the lack of formal powering, exploratory tests were performed to compare the study groups. Between-arm comparisons were made under the intention-to-treat framework. Given the clustered nature of data derived from the group participants, a partially clustered model (modelling the clustering in the intervention arm) using a linear mixed effects model was estimated. This was undertaken using restricted maximum likelihood with a Kenward-Roger degrees of freedom correction (recommended for small sample size and/or small number of clusters) on treatment allocation. The estimate was adjusted for baseline value of the outcome and site, with a random intercept for each group in the intervention arm and one intercept for the control arm (i.e. partial clustering); this also allowed for heteroskedastic errors between treatments arms. Results are presented as adjusted difference in means alongside associated 95% CI; the treatment effect estimate is also presented as a standardised effect size for completeness.

A reflexive thematic approach was used to analyse the qualitative data because of its flexibility and pragmatism in helping us to derive an understanding of the range of experiences and responses to the interventions and study procedures. A preliminary deductive coding framework was developed based on concepts discussed in the interview topic guide. Sub-codes were then derived inductively through an open coding of the content of each area. Themes and subthemes were regularly presented to the senior qualitative researcher (L.B.) and chief investigator (P.M.) to ensure the emerging analysis was trustworthy and credible. The framework was refined, with coded material regrouped, as new data from subsequent interviews was gathered and a deeper level of understanding achieved.

The cost of delivering perinatal ESGs was calculated as the product of applicable healthcare unit cost(s)^31^ and staff time involved. To determine the feasibility of data collection of the resource use components, we explored the availability of data from perinatal mental health team records, as well as the completeness of resource use questionnaire data.

### Ethics

The authors assert that all procedures contributing to this work comply with the ethical standards of the relevant national and institutional committees on human experimentation and with the Helsinki Declaration of 1975, as revised in 2013. All procedures involving patients were approved by Camden & Kings Cross Research Ethics Committee (Reference 21/LO/0833).

## Transparency declaration

P.M. is the manuscript guarantor and affirms that the manuscript is an honest, accurate and transparent account of the study being reported and that no important aspects of the study have been omitted.

## Results

**Figure 1:**
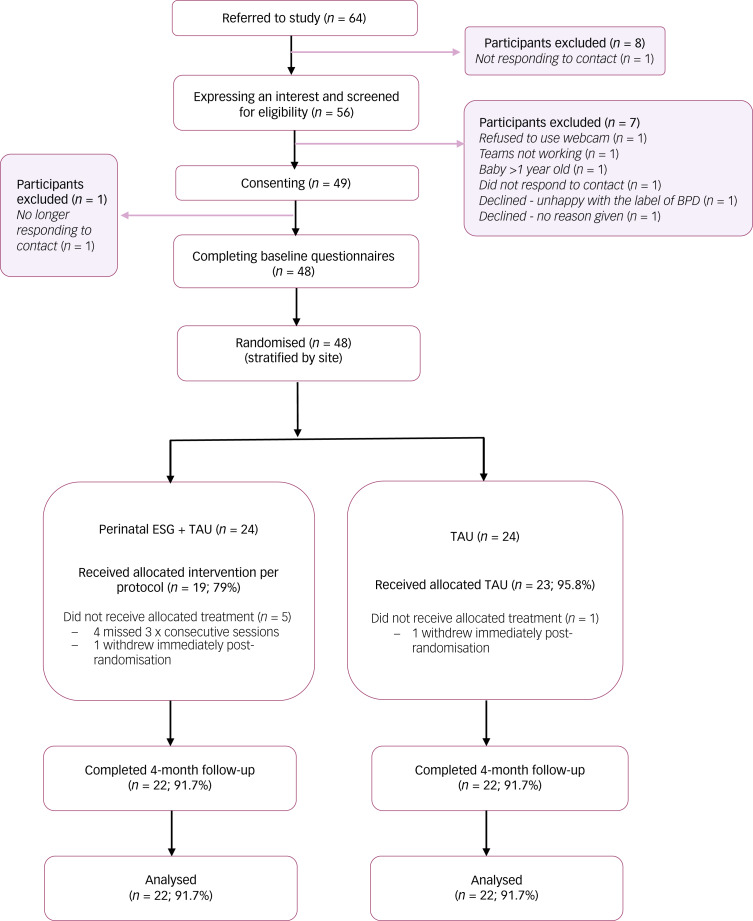
CONSORT diagram capturing participant flow. BPD, borderline personality disorder; CONSORT, Consolidated Standards of Reporting Trials; ESG, emotional skills group.

Between 15 September 2022 and 29 March 2023, 64 people were referred into the study, of whom 48 people were deemed eligible and consented to participate, equating to a consent rate of 75%. Of the 48 who met eligibility criteria and were randomised, 24 were allocated to perinatal ESGs + TAU, and 24 to TAU alone. Four months post randomisation, 92% of participants in both arms completed the 4-month follow-up assessment. A CONSORT diagram displaying participant flow is displayed in Fig. 1.

Baseline characteristics of the recruited sample are displayed in Table 1. The trial's arms were well balanced on participant characteristics. The majority of participants (71%) were White; 17% were from mixed/multiple ethnic groups, 10% were Black, and 2% reported being from another ethnic group. The median number of BPD symptoms was 9 (where the maximum score is 9) and median CORE-10 score was 25, corresponding to moderately severe levels of emotional distress.

**Table 1:** Baseline characteristics

	Intervention (*N* = 24)	Control (*N* = 24)	Total (*N* = 48)
Age (years, s.d.)	29 (4)	30 (6)	29 (5)
Relationship status:			
Married/civil partnership	5 (21%)	4 (17%)	9 (19%)
Cohabiting (living with partner)	5 (21%)	9 (38%)	14 (29%)
Spouse/partner deceased	0	1 (4%)	1 (2%)
Separated	1 (4%)	1 (4%)	2 (4%)
Single /non-cohabiting partner	12 (50%)	8 (33%)	20 (42%)
Other	1 (4%)	1 (4%)	2 (4%)
Current living arrangements:			
Alone	1 (4%)	1 (4%)	2 (4%)
With spouse/partner	3 (13%)	1 (4%)	4 (8%)
With spouse/partner and child or children	7 (29%)	11 (46%)	18 (38%)
With child or children (but no spouse/partner)	11 (46%)	8 (33%)	19 (40%)
With other relatives	1 (4%)	1 (4%)	2 (4%)
None of the above	1 (4%)	2 (8%)	3 (6%)
Ethnicity:			
Asian/Asian British	0	0	0
Black/African/Caribbean/Black British	3 (13%)	2 (8%)	5 (10%)
Mixed/Multiple ethnic groups	3 (13%)	5 (21%)	8 (17%)
White	17 (71%)	17 (71%)	34 (71%)
Other ethnic group	1 (4%)	0	1 (2%)
Current work situation:			
In paid work (including self-employed)	12 (50%)	13 (54%)	25 (52%)
Unemployed	5 (21%)	2 (8%)	7 (15%)
Permanently sick or disabled	1 (4%)	2 (8%)	3 (6%)
Looking after home or family	4 (17%)	6 (25%)	10 (21%)
Full-time student	1 (4%)	0	1 (2%)
Other	1 (4%)	1 (4%)	2 (4%)
Pregnancy status at recruitment:			
Currently pregnant	9 (38%)	9 (38%)	18 (38%)
Given birth in the last year	15 (63%)	15 (63%)	30 (63%)
Centre:			
South West	12 (50%)	12 (50%)	24 (50%)
London	12 (50%)	12 (50%)	24 (50%)
SAPAS (median, IQR)	6 [5–7]	7 [6.5–7.5]	7 [5–7]
BPD score (median, IQR)	9 [8–9]	9 [8–9]	9 [8–9]
ZAN-BPD-SR (mean, SD)	15.3 (7.3)	17.8 (6.2)	16.5 (6.8)
CORE-10 (median, IQR)	25 [19–26]	26 [22–27]	25 [20–27]
PSS (median, IQR)^a^	53 [40–61]	47 [39–56]	50 [40–60]
Episodes of self-harm in the past week:			
Yes, once	0	2 (8%)	2 (4%)
Yes, more than once	0	0	0
No	24 (100%)	22 (92%)	46 (96%)
SWEMWBS (mean, SD)	17.2 (3.7)	16.8 (1.8)	17.0 (8.5)
WSAS (median, IQR)	25 [20–31]	28 [22–30]	25 [20–30]
EQ-5D-5L index value (median, IQR)	0.78 [0.56–0.91]	0.71 [0.45–0.84]	0.75 [0.53–0.87]
EQ-5D VAS (mean, SD)	47 (24)	50 (25)	49 (24)

SAPAS, Standardised Assessment of Personality – Abbreviated Scale; IQR, interquartile range; BPD, borderline personality disorder; ZAN-BPD-SR, Zanarini Rating Scale for Borderline Personality Disorder Self-Report Scale; CORE-10, Ten-item Clinical Outcomes in Routine Evaluation Scale; PSS, Parenting Stress Scale; SWEMWBS, Short Warwick Edinburgh Mental Wellbeing Scale; WSAS, Work and Social Adjustment Scale; EQ-5D-5L, EuroQol-5 Dimensions-5 Levels; EQ-5D VAS, EuroQol-5 Dimensions Visual Analogue Scale.

a. *n* = 30, 18/48 were pregnant at recruitment.

For those randomised to the intervention arm, the median number of perinatal ESG sessions attended was nine (interquartile range 6–11). In all, 3/24 (13%) participants allocated to the intervention attended all 12 sessions (i.e. 100% of the course). A total of 13/24 (54%) participants allocated to the intervention attended at least nine sessions (i.e. 75% of the course) and 19/24 (79%) participants allocated to the intervention attended at least six sessions (i.e. 50% of the course). Two participants attended no sessions, and of these one withdrew from the study after randomisation; one was withdrawn from the intervention after non-attendance of the first three sessions. Three participants were withdrawn from the intervention because they did not attend three consecutive sessions (as per treatment protocol). Where recorded, a range of practical reasons were given for missed sessions, including giving birth, baby being ill and having to attend a general practitioner (GP) or hospital appointment. The reasons provided did not indicate that non-attendance was related to the unacceptability of the trial or intervention, nor because of perceived ineffectiveness (or effectiveness) of the intervention.

Table 2 presents 2- and 4-month follow-up scores. At both 2- and 4-month follow-up, levels of BPD symptoms and emotional distress were lower among those allocated to perinatal ESGs.

**Table 2:** 2- and 4-month outcome findings

Outcome	2 months post randomisation		4 months post randomisation	
	Intervention	Control	Intervention	Control
ZAN-BPD-SR	11.3 (5.2)	13.8 (6.4)	8.7 (5.9)	12.0 (6.8)
CORE-10	19.5 [15–24]	20 [15–23]	15 [12–19]	20 [14–24]
PSS	46.9 (11.2)^a^	44.1 (10.1)^a^	44.9 (10.6)^b^	42.1 (7.3)^b^
SWEMWBS	19.6 [17.4–20.7]	19.3 [16.9–20.7]	20.4 [18.0–22.4]	18.6 [17.4–21.5]
WSAS	21.8 (7.4)	25.3 (8.9)	21 (11.1)	23 (8.3)
EQ-5D-5L index value	−	−	0.87 [0.74–0.92]	0.85 [0.68–0.92]
EQ−5D VAS	−	−	57 (22)	59 (17)

ZAN-BPD-SR, Zanarini Rating Scale for Borderline Personality Disorder Self-Report Scale; CORE-10, Ten-item Clinical Outcomes in Routine Evaluation Scale; PSS, Parenting Stress Scale; SWEMWBS, Short Warwick Edinburgh Mental Wellbeing Scale; WSAS, Work and Social Adjustment Scale; EQ-5D-5L, EuroQol-5 Dimensions-5 Levels; EQ-5D VAS, EuroQol-5 Dimensions Visual Analogue Scale.

a. *n* = 28.

b. *n* = 41.

Table 3 displays the results from regression analyses of the 4-month follow-up data.

**Table 3:** Results from regression analyses of 4-month outcome data

	Coefficient (95% CI)	Point estimate as standardised effect size
ZAN-BPD-SR	−2.0 (−6.2, 2.1)	0.3
CORE-10	−2.4 (−6.2, 1.5)	0.4
PSS^a^ (*n* = 27)	1.7 (−6.6, 10.0)	0.1
PSS (*n* = 41)	2.7 (−5.0, 10.4)	0.2
SWEMWBS	0.6 (−1.2, 2.4)	0.1
WSAS	−1.5 (−7.8, 4.9)	0.2
EQ-5D-5L index value	0.02 (−0.10, 0.15)	0.1
EQ-5D VAS	−1.6 (−16.2, 13.1)	0.1

Coefficients represent adjusted difference in means.

ZAN-BPD-SR, Zanarini Rating Scale for Borderline Personality Disorder Self-Report Scale; CORE-10, Ten-item Clinical Outcomes in Routine Evaluation Scale; PSS, Parenting Stress Scale; SWEMWBS, Short Warwick Edinburgh Mental Wellbeing Scale; WSAS, Work and Social Adjustment Scale; EQ-5D-5L, EuroQol-5 Dimensions-5 Levels; EQ-5D VAS, EuroQol-5 Dimensions Visual Analogue Scale.

a. Restricted to those with baseline data.

The coefficients represent the average effect of the intervention on outcome domains at end of treatment. In the case of the ZAN-BPD-SR, the coefficient of −2.0 indicates that on average, at 4 months post randomisation, the ZAN-BPD scores of those allocated to perinatal ESGs were 2 points less than those in the control arm. Putting this into a clinical context, the minimum clinically important difference (MCID) for the ZAN-BPD-SR is a 3.5-point reduction in score.^32^ The direction of the point estimate of the proposed primary outcome (ZAN-BPD-SR) favoured the intervention; the upper bound of the 95% CI included the MCID for treatment effectiveness, while the lower bound excluded an MCID favouring control. This was consistent across other clinical outcomes, whereby the direction (CORE-10, WEMWBS and WSAS) favoured the intervention. The health-related quality of life measures were equivocal with the direction of the EQ-5D-5L favouring the intervention, whereas the EQ-5D VAS favoured the control. The confidence intervals of all the estimates crossed the null value, indicating that none of the findings were statistically significant at the 5% level.

Three serious adverse events were recorded during the trial – two in the intervention arm and one in the control arm. All the events were hospital admissions for physical or mental health conditions, and none of these were thought to be related to the trial.

The estimated average cost of delivering perinatal ESGs was £918 per participant; this is in addition to any costs associated with providing TAU. Completeness of resource data collection was high: from 100% of participants at baseline and 91% of participants at 4 months. The data sources indicated that as well as perinatal mental health contacts, community health (GP, health visitor, midwifery) and mental health prescribing all contributed to costs and should be collected in any future economic evaluation.

The qualitative data provided further support for the positive impact of perinatal ESGs on improving the mental health and social functioning of perinatal people with BPD. All the interviewed participants allocated to the intervention arm reported that the perinatal ESGs had made a positive impact on their mental health. In particular, they expressed feeling better equipped with skills to navigate relationships and regulate their emotions:

‘A massive impact. I mean, I am completely different I think to how I was before. I have said to so many different people, like the whole perinatal team has helped me loads, but this group has given me so many tools to deal with different … so many different things. It's not like it's sort of just touched the surface with one thing I was struggling with – it has made a huge difference to me and I am just so much happier.’ (SW-029)

Some reported that the skills learned during the groups had helped them to better express their needs:

‘It's definitely helping my family life. So, in terms of communicating with my husband as to how I am feeling, if I need help with my daughter or if I am struggling with particular thoughts, it's helped me kind of one, articulate that better and two, helping me manage those feelings. So, by communicating with him I can then give myself time properly to do what I need to do to get myself back and stable.’ (SW-031)

Many of the participants also reported deriving benefit from being part of a group that helped them to ‘realise I'm not alone out there’ (LO-009) and identify with others experiencing similar problems who made them ‘feel like I wasn't crazy’ (SW-023).

Most of the participants also reported that being in a mixed perinatal group (i.e. with both pregnant and postpartum people) was a positive experience because those who had already delivered their baby could support their pregnant peers by sharing advice and experiences. All participants reported that the online delivery of the group was acceptable and appropriate and without major technical issues. Although one participant expressed a preference for face-to-face group delivery, they also mentioned that maintaining regular attendance might have been more challenging in that format.

Staff delivering the groups gave positive feedback about the supervision and training they had received and reported finding the consultation meetings with other staff particularly helpful. Although the online delivery of treatment was convenient for everyone and helped to optimise attendance, some staff felt that it could sometimes give sessions a ‘podcast feel’ which needed active management to prevent people from not fully participating. Regarding the session duration, all interviewees agreed 2 h was about right and that 12 sessions fitted conveniently into school terms (which was a concern for participants with school-age children).

All staff spoke of the positive impact of perinatal ESGs on participants’ mental health:

‘We had one person who said that ‘check the facts’ [a modular component of ESGs] has been, like, lifechanging for her, and, you know, that she does that all the time now. She questions whether she's interpreting a situation with judgements or evaluations that get in the way for her. And, like, she just looked brighter. She'd started going to mum and baby groups. Yeah, I think she made a lot of progress.’ (SW-Staff-2)

Participants and staff all reported that the study procedures had been acceptable. Although some TAU participants expressed feeling initially disappointed with their assigned group and another felt relief for not being allocated to the ESG (which she would have withdrawn from), most of the participants reported finding the randomisation process and communication acceptable and clear:

‘I think I like that it's random. Because it kind of feels a bit more equal, if it's just completely random. I think that's a good thing.’ (LO-003)

## Discussion

### Main findings

Our overarching aim was to investigate whether it is feasible to undertake a randomised controlled trial of perinatal ESGs for people with BPD. Our results demonstrate that such a trial is entirely feasible. We recruited 100% of our pre-specified target and followed-up 92% of all participants at 4 months. In the intervention group, 54% of participants attended 75% of the 12 sessions, which was below our 75% target. Notwithstanding, this was a high bar for determining adherence, and nearly 80% of participants attended at least half of the group treatment, with high median attendance of nine sessions (equating to 75% of the 12-session group course). The trial steering committee overseeing this study reported that although performance on this criterion fell within the ‘review’ category, for a perinatal sample of people with severe symptoms of BPD this represented a very positive achievement indeed.

There were no statistically significant changes in any of the patient-reported outcome measures at 4 months post randomisation. However, the study was a feasibility trial that was not powered to detect statistically significant differences. Furthermore, the direction of point estimate for the proposed primary outcome (ZAN-BPD-SR total score) favoured the intervention and importantly, the 95% CI included the clinically important difference for this measure. This finding was consistent across other patient-rated clinical outcome measures, which also favoured the intervention. We were also able to demonstrate that a future economic evaluation appears feasible. From a qualitative perspective, many of the trial participants spoke favourably about benefits gained in their mental health and family life that they had achieved through the perinatal ESGs. The online delivery was acceptable to participants and optimised attendance during a very busy period in their lives. In addition, the heterogenous make-up of groups (consisting of both pregnant and non-pregnant people) allowed those in the antenatal phase to learn from those in the postnatal phase. We purposefully ran the groups with both antenatal and postnatal people for several reasons. First, the inclusion of antenatal and postnatal participants within the same group reflects the reality of NHS service delivery where both pregnant and postnatal people are treated within the same perinatal mental health team. Second, prior observational work^20^ shows that it is acceptable to run groups with both pregnant and postnatal people, and that most people do not view pregnancy and the postnatal period as ‘separate’ parts of their perinatal experience. Notwithstanding, it is possible that the perinatal stage affects the benefits and learning experiences derived from ESGs, and future research should address this through, for example, subgroup analyses of trial populations.

### Methodological considerations

In terms of strengths, we recruited a sample with high external validity to populations of people with BPD seen by specialist perinatal community mental health teams. We adopted broad inclusion criteria and a limited number of exclusion criteria. The median CORE-10 score was 25, corresponding to moderately severe levels of emotional distress, and the mean mental well-being score (17; s.d. 8.5) was below the UK general population mean of 23.5 (s.d. 3.9).^25^ The sample also had functional impairment linked to moderately severe psychopathology.^26^ We recruited 100% of our pre-specified target and obtained post-treatment outcome data on 92% of participants. Taking into account the small sample size, the participant characteristics were well balanced between trial arms.

In terms of limitations, to meet the feasibility study objectives, the length of follow-up was limited to 4 months. However, BPD is a long-term mental health condition, and it is important to investigate the long-term effect of interventions on the condition. Moreover, a definitive trial should investigate the longer-term impacts of perinatal ESGs on birthing parents and the relationship with their babies. In keeping with the approach adopted by other recent trials in the personality disorder field,^33,34^ we did not include a detailed assessment of personality disorders. Such assessments are time-consuming to complete and not in keeping with the needs of a pragmatic study conducted in a busy clinical setting. Finally, the apparent benefits of perinatal ESGs may have been because of non-specific factors related to belonging to a group which we did not measure or adjust for. Any future study should investigate the moderating effects of group dynamics and therapeutic alliance, as well as the mechanism of action of perinatal ESGs.

### Implications

Our study has shown that it is feasible and acceptable to conduct a clinical trial of perinatal ESGs for women and birthing people with BPD. Furthermore the quantitative and qualitative findings suggest that perinatal ESGs may have promising effects on mental health and social functioning, and that the efficacy of perinatal ESGs should be tested in a fully powered trial.

Ensuring that all birthing parents and their babies receive timely, more effective and accessible perinatal mental health interventions is critical because untreated perinatal mental health issues have serious long-term adverse consequences for both the parent and the child.^35^ Although ESGs constitute a brief intervention for people with complex needs associated with BPD, observational data indicate that brief treatment for BPD may be effective.^36^ Furthermore, trials of brief interventions for people with BPD are scarce, and there is a pressing need to enhance the evidence base in this field.^33^ Ultimately, if people with BPD could be effectively helped during the perinatal period, they might be able to practise more effective ways of managing their emotions and relationships before their babies are exposed to the full range of environmental risks. In light of this, the NHS is now committed to ‘increasing access to evidence-based care for women with moderate to severe perinatal mental health difficulties and a personality disorder diagnosis, to benefit an additional 24 000 women per year by 2023/24’.^37^ Yet, there is a conspicuous lack evidence to inform the roll-out of plans such as those proposed for the NHS. In this respect, determining the efficacy of perinatal ESGs for women and birthing people with BPD should be a research priority, as it could provide crucial insights for improving mental health services across the world for this previously neglected population.

## Data Availability

The data that support the findings of this study are available from the corresponding author, P.M., upon reasonable request.

## References

[1] Chesney E, Goodwin GM, Fazel S. Risks of all-cause and suicide mortality in mental disorders: a meta-review. World Psychiatry 2014; 13(2): 153–60. 24890068 10.1002/wps.20128 PMC4102288

[2] Pare-Miron V, Czuzoj-Shulman N, Oddy L, Spence AR, Abenhaim HA. Effect of borderline personality disorder on obstetrical and neonatal outcomes. Womens Health Issues 2016; 26(2): 190–5. 26718528 10.1016/j.whi.2015.11.001

[3] Florange JG, Herpertz SC. Parenting in patients with borderline personality disorder, sequelae for the offspring and approaches to treatment and prevention. Curr Psychiatry Rep 2019; 21(2): 9. 30729325 10.1007/s11920-019-0996-1

[4] Eyden J, Winsper C, Wolke D, Broome MR, MacCallum F. A systematic review of the parenting and outcomes experienced by offspring of mothers with borderline personality pathology: potential mechanisms and clinical implications. Clin Psychol Rev 2016; 47: 85–105. 27261413 10.1016/j.cpr.2016.04.002

[5] Conroy S, Pariante CM, Marks MN, Davies HA, Farrelly S, Schacht R, et al. Maternal psychopathology and infant development at 18 months: the impact of maternal personality disorder and depression. J Am Acad Child Adolesc Psychiatry 2012; 51(1): 51–61. 22176939 10.1016/j.jaac.2011.10.007

[6] Rutter M, Quinton D. Parental psychiatric disorder: effects on children. Psychol Med 1984; 14(4): 853–80. 6545419 10.1017/s0033291700019838

[7] Howard LM, Molyneaux E, Dennis C-L, Rochat T, Stein A, Milgrom J. Non-psychotic mental disorders in the perinatal period. Lancet 2014; 384(9956): 1775–88. 25455248 10.1016/S0140-6736(14)61276-9

[8] Royal College of Psychiatrists. Perinatal Specialist Community Mental Health Team Service Specification Template. Royal College of Psychiatrists, 2018 (https://www.rcpsych.ac.uk/docs/default-source/improving-care/nccmh/perinatal/nccmh-perinatal-specialist-community-mental-health-team-service-spec-template-may2018.pdf?sfvrsn=aa70cd14_4).

[9] Crowley G, Molyneaux E, Nath S, Trevillion K, Moran P, Howard LM. Disordered personality traits and psychiatric morbidity in pregnancy: a population-based study. Arch Womens Ment Health 2019; 23: 43–52. 30612198 10.1007/s00737-018-0937-8 PMC6987086

[10] Storebø OJ, Stoffers-Winterling JM, Völlm BA, Kongerslev MT, Mattivi JT, Jørgensen MS, et al. Psychological therapies for people with borderline personality disorder. Cochrane Database of Syst Rev 2020; 5(5): CD012955. 32368793 10.1002/14651858.CD012955.pub2 PMC7199382

[11] Iliakis EA, Sonley AK, Ilagan GS, Choi-Kain LW. Treatment of borderline personality disorder: is supply adequate to meet public health needs? Psychiatr Serv 2019; 70(9): 772–81. 31138059 10.1176/appi.ps.201900073

[12] Stoffers-Winterling JM, Storebo OJ, Kongerslev MT, Faltinsen E, Todorovac A, Sedoc Jorgensen M, et al. Psychotherapies for borderline personality disorder: a focused systematic review and meta-analysis. Br J Psychiatry 2022; 221(3): 538–52. 35088687 10.1192/bjp.2021.204

[13] Muzik M, Rosenblum KL, Alfafara EA, Schuster MM, Miller NM, Waddell RM, et al. Mom power: preliminary outcomes of a group intervention to improve mental health and parenting among high-risk mothers. Arch Womens Ment Health 2015; 18(3): 507–21. 25577336 10.1007/s00737-014-0490-z PMC4439267

[14] Hellberg SN, Bruening AB, Thompson KA, Hopkins TA. Applications of dialectical behavioural therapy in the perinatal period: a scoping review. Clin Psychol Psychother 2024; 31(1): e2937. 10.1002/cpp.2937 38116846

[15] Liu Y, Pencheon E, Hunter RM, Moncrieff J, Freemantle N. Recruitment and retention strategies in mental health trials – a systematic review. PLoS One 2018; 13(8): e0203127. 30157250 10.1371/journal.pone.0203127 PMC6114918

[16] Moran P, Bick D, Biddle L, Borries B, Kandiyali R, Rigby J, et al. A feasibility randomised controlled trial with an embedded qualitative evaluation of perinatal emotional skills groups for women with borderline personality disorder: protocol for the EASE study. Pilot Feasibility Stud 2022; 8(1): 215. 36151584 10.1186/s40814-022-01177-y PMC9503265

[17] Moran P, Leese M, Lee T, Walters P, Thornicroft G, Mann A. Standardised assessment of personality – abbreviated scale (SAPAS): preliminary validation of a brief screen for personality disorder. BrJ Psychiatry 2003; 183: 228–32. 12948996 10.1192/bjp.183.3.228

[18] McManus S, Bebbington P, Jenkins R, Brugha T, eds. Mental Health and Wellbeing in England: Adult Psychiatric Morbidity Survey 2014. NHS Digital, 2016.

[19] American Psychiatric Association. Diagnostic and Statistical Manual of Mental Disorders (DSM-5®). American Psychiatric Association, 2013.

[20] Wilson H, Donachie AL. Evaluating the effectiveness of a dialectical behaviour therapy (DBT) informed programme in a community perinatal team. Behav Cogn Psychother 2018; 46(5): 541–53. 29366433 10.1017/S1352465817000790

[21] British Psychological Society and Royal College of Psychiatrists. Antenatal and Postnatal Mental Health: Clinical Management and Service Guidance. British Psychological Society and Royal College of Psychiatrists, 2014.

[22] Zanarini MC, Weingeroff JL, Frankenburg FR, Fitzmaurice GM. Development of the self-report version of the Zanarini rating scale for borderline personality disorder. Personal Ment Health 2015; 9(4): 243–9. 26174588 10.1002/pmh.1302 PMC4609276

[23] Barkham M, Bewick B, Mullin T, Gilbody S, Connell J, Cahill J, et al. The CORE-10: a short measure of psychological distress for routine use in the psychological therapies. Couns Psychother Res 2013; 13(1): 3–13.

[24] van Asselt AD, Dirksen CD, Arntz A, Giesen-Bloo JH, Severens JL. The EQ-5D: a useful quality of life measure in borderline personality disorder? Eur Psychiatry 2009; 24(2): 79–85. 19095421 10.1016/j.eurpsy.2008.11.001

[25] Fat LN, Scholes S, Boniface S, Mindell J, Stewart-Brown S. Evaluating and establishing national norms for mental wellbeing using the Short Warwick-Edinburgh Mental Well-Being Scale (SWEMWBS): findings from the health survey for England. Qual Life Res 2017; 26(5): 1129–44. 27853963 10.1007/s11136-016-1454-8 PMC5376387

[26] Mundt JC, Marks IM, Shear MK, Greist JH. The work and social adjustment scale: a simple measure of impairment in functioning. Br J Psychiatry 2002; 180: 461–4. 11983645 10.1192/bjp.180.5.461

[27] Berry J, Jones W. The parental stress scale : initial psychometric evidence. J Soc Pers Relat 1995; 12: 463–72.

[28] Madge N, Hewitt A, Hawton K, de Wilde EJ, Corcoran P, Fekete S, et al. Deliberate self-harm within an international community sample of young people: comparative findings from the Child & Adolescent Self-harm in Europe (CASE) study. J Child Psychol Psychiatry 2008; 49(6): 667–77. 18341543 10.1111/j.1469-7610.2008.01879.x

[29] Lancaster GA, Dodd S, Williamson PR. Design and analysis of pilot studies: recommendations for good practice. J Eval Clin Pract 2004; 10(2): 307–12. 15189396 10.1111/j..2002.384.doc.x

[30] Eldridge SM, Chan CL, Campbell MJ, Bond CM, Hopewell S, Thabane L, et al. CONSORT 2010 statement: extension to randomised pilot and feasibility trials. Pilot Feasibility Stud 2016; 2: 64. 27965879 10.1186/s40814-016-0105-8 PMC5154046

[31] Jones K, Weatherly H, Birch S, Castelli A, Chalkley M, Dargan A, et al. *Unit Costs of Health and Social Care 2022 Manual Kent*. Kent Academic Repository, 2022 (https://kar.kent.ac.uk/100519/1/Unit%20Costs%20of%20health%20and%20Social%20Care%202022%20%28amended%2013%20July%202023%29.pdf).

[32] Juul S, Simonsen S, Poulsen S, Lunn S, Sorensen P, Bateman A, et al. Detailed statistical analysis plan for the short-term versus long-term mentalisation-based therapy for outpatients with subthreshold or diagnosed borderline personality disorder randomised clinical trial (MBT-RCT). Trials 2021; 22(1): 497. 34321051 10.1186/s13063-021-05450-y PMC8316699

[33] Crawford MJ, Thana L, Parker J, Turner O, Carney A, McMurran M, et al. Structured psychological support for people with personality disorder: feasibility randomised controlled trial of a low-intensity intervention. BJPsych Open 2020; 6(2): e25. 32115015 10.1192/bjo.2020.7 PMC7176836

[34] Day C, Briskman J, Crawford MJ, Foote L, Harris L, Boadu J, et al. Randomised feasibility trial of the helping families programme-modified: an intensive parenting intervention for parents affected by severe personality difficulties. BMJ Open 2020; 10(2): e033637. 10.1136/bmjopen-2019-033637 PMC7045220 32034024

[35] NICE Clinical Guideline, CG 192. *Antenatal and Postnatal Mental Health: Clinical Management and Service Guidance*. NICE, 2020.

[36] Laporte L, Paris J, Bergevin T, Fraser R, Cardin JF. Clinical outcomes of a stepped care program for borderline personality disorder. Personal Ment Health 2018; 12(3): 252–64. 29709109 10.1002/pmh.1421

[37] Department of Health and Social Care. The NHS Long Term Plan. Department of Health and Social Care, 2019.

